# Visible T1-hyperintensity of the dentate nucleus after multiple administrations of macrocyclic gadolinium-based contrast agents: yes or no?

**DOI:** 10.1186/s13244-019-0767-x

**Published:** 2019-09-04

**Authors:** Alessandra Splendiani, Antonella Corridore, Silvia Torlone, Milvia Martino, Antonio Barile, Ernesto Di Cesare, Carlo Masciocchi

**Affiliations:** Department of Biotechnological and Applied Clinical Sciences, University of L’Aquila, 67100 L’Aquila, Italy

**Keywords:** Gadolinium-based contrast agents, Gadolinium retention, Dentate nucleus, Gadoterate dimeglumine, Gadobutrol

## Abstract

**Objectives:**

To investigate the appearance of visible dentate nucleus (DN) T1-hyperintensity and quantify changes in DN/pons (DN/P) signal intensity (SI) ratio in MS patients after the exclusive administration of macrocyclic GBCAs.

**Materials and methods:**

One hundred forty-nine patients with confirmed MS were evaluated. Patients received at least two administrations of gadobutrol (*n* = 63), gadoterate (*n* = 57), or both (*n* = 29). Two experienced neuroradiologists in consensus evaluated unenhanced T1-weighted MR images from all examinations in each patient for evidence of visible DN hyperintensity. Thereafter, SI measurements were made in the left and right DN and pons on unenhanced T1-weighted images from the first and last scans. A two-sample *t* test compared the DN/P SI ratios for patients with and without visible T1-hyperintensity.

**Results:**

Visible T1-hyperintensity was observed in 42/149 (28.2%) patients (19 after gadobutrol only, 15 after gadoterate only, 8 after both), typically at the 4th or 5th follow-up exam at 3–4 years after the initial examination. Significant increases in DN/P SI ratio from first to last examination were determined for patients with visible T1-hyperintensity (0.998 ± 0.002 to 1.153 ± 0.016, *p* < 0.0001 for gadobutrol; 1.003 ± 0.004 to 1.110 ± 0.014, *p* < 0.0001 for gadoterate; 1.004 ± 0.011 to 1.163 ± 0.032, *p* = 0.0004 for both) but not for patients without visible T1-hyperintensity (*p* > 0.05; all groups).

**Conclusion:**

Multiple injections of gadobutrol and/or gadoterate can lead to visible and quantifiable increases in DN/P SI ratio in some patients with MS.

## Key points

- Visible T1-hyperintensity of the dentate nucleus on unenhanced T1-weighted images of MS patients can occur after the exclusive administration of the macrocyclic GBCAs Dotarem and Gadovist.
- Visible T1-hyperintensity is associated with significant increases in DN/P SI ratio.
- 11Variation of changes in DN/P SI ration across patients suggests the appearance of visible T1-hyperintensity is a patient-specific process rather than a GBCA-specific process.
- There is no evidence that the appearance of visible T1-hyperintensity or changes in DN/P SI ratio has any impact on patient health or safety.

## Introduction

High signal intensity (SI) in the dentate nucleus (DN) and globus pallidus (GP) on unenhanced T1-weighted MR images following the cumulative administration of exclusively macrocyclic gadolinium-based contrast agents (GBCAs) is currently a source of debate and controversy. The widely held view is that visible T1-hyperintensity occurs primarily after the cumulative administration of so-called linear GBCAs rather than macrocyclic GBCAs and that the observed high T1-signal possibly reflects the release of gadolinium (Gd) from the linear chelating molecule and its subsequent retention in the brain, possibly through binding to cellular macromolecules [1–6]. Although no clinical signs or symptoms attributable to brain Gd retention have yet been observed after cumulative exposure to any GBCA, linear or macrocyclic [7–11], the widespread assumption is that retained Gd presumed to have been released from linear GBCAs and “visible” on unenhanced T1-weighted images potentially poses a long-term risk to human health [12] while the absence of manifest T1-signal increases with macrocyclic GBCAs is taken as indicating their inherent “safety”. A consequence of this assumption in Europe has been the suspension or restriction of certain linear GBCAs for clinical use [12].

In recent years, numerous studies have demonstrated T1-signal increases in the brain after the exclusive administration of macrocyclic GBCAs [13–19]. Invariably, however, these studies have been criticized and marginalized, often by the manufacturers of the GBCA in question [20–23], in part, because the images presented do not show obvious T1-hyperintensity of the DN or GP to the extent seen with linear GBCAs [20]. Unfortunately, such criticism overlooks the fact that brain Gd retention has unequivocally been demonstrated not only in myriad animal studies [24–31] but also in human brain autopsy samples after the exclusive administration of macrocyclic GBCAs [32].

Patients with relapsing-remitting multiple sclerosis (RR-MS) typically undergo frequent GBCA-enhanced MR studies as part of their routine clinical follow-up. Studies looking at T1-signal changes in the DN and/or GP of patients with RR-MS after the exclusive serial administration of macrocyclic GBCAs have yielded conflicting results. Whereas some studies have failed to observe T1-signal changes [33–38], others have noted marked quantitative changes [13, 17]. Recently, Splendiani et al. [15] reported visible T1-hyperintensity in the DN in roughly one third of patients with RR-MS who had received cumulative doses of the macrocyclic GBCAs gadobutrol and/or gadoterate. The aim of our study was to further investigate patients with RR-MS for the presence of visible signal enhancement of the DN on unenhanced T1-weighted images following multiple exposures to macrocyclic GBCAs.

## Methods and materials

### Patients

This single-center retrospective study was approved by the institutional review board and the need for patient informed consent was waived. We reviewed our PACS database for all patients with confirmed RR-MS who underwent two or more MRI exams exclusively at our center with the macrocyclic GBCAs gadoterate meglumine (Dotarem; Guerbet, Aulnay-sous-Bois, France) and/or gadobutrol (Gadovist; Bayer Healthcare, Berlin, Germany) between January 2005 and December 2017. Patients were included only if complete documentation regarding contrast administration (i.e., date of exam, type, and dose of GBCA administered) was available for each MRI exam. Patients were ineligible for inclusion if they had an eGFR of ≤ 60 mL/min/1.73 m^2^ based on a blood sample taken close to the date of the last MR examination; a history of cerebrovascular events including hemorrhage, stroke, or brain ischemia; previous cerebral neurosurgical treatments or radiation therapy; intra-axial brain tumor or other lesions such as vascular malformations located in the cerebellum or pons; a history of intracranial infection, such as meningitis or encephalitis; abnormal liver function or hepatic deficiency. Abnormal liver function was defined as the alteration of one or more of serum parameters as alanine aminotransferase, γ-glutamyl transpeptidase, or aspartate aminotransferase. Hepatic deficiency was considered an exclusion criterion as it can modify GBCA pharmacokinetics and because high signal intensity in basal ganglia on T1-weighted images is associated with hepatic cirrhosis and other forms of liver disease [39, 40].

For all included patients the age, sex, number of GBCA administrations, and accumulated dose were recorded. An estimation of the mean interval (± standard deviation) between GBCA administrations was calculated by dividing the time in weeks between the first and last MRI examinations by the total number of GBCA administrations made during that period.

### MRI protocol

All MRI exams were performed on a 3 T scanner (GE Healthcare-Signa EXCITE). Axial T1-weighted spin-echo images were acquired before and after GBCA injection with repetition time (TR) = 500–588 ms, echo time (TE) = 7.7–12 ms, matrix = 256 × 256, section thickness = 4 mm, and field of view (FOV) = 250 mm. Axial T2-weighted images were acquired with TR = 4890 ms, TE = 85 ms, section thickness = 4 mm, matrix = 256 × 256 and FOV = 250 mm. Axial susceptibility weighted images (SWI) were acquired with TR/TE = 85.2/32.6 ms, a slab of 64 slices of 3 mm thickness, no gap, FOV = 220 mm, an acquisition matrix of 384 × 256, number of excitations (NEX) = 2, and a flip angle of 20°. Fluid-attenuated inversion recovery (FLAIR) and T1-weighted non-contrast sequences were used to precisely identify the DN and verify visible T1-hyperintensity after multiple contrast administrations.

All patients received a standardized dose of 0.1 mmol/kg body weight of either gadoterate or gadobutrol for each examination.

### Data collection

Two neuroradiologists in consensus (AS and AC with 25 and 4 years’ experience, respectively) evaluated unenhanced T1-weighted images from each MRI examination in each patient for visible T1-hyperintensity in the DN. Patients without visible T1-hyperintensity on any image were considered negative whereas patients with clearly discernable T1-hyperintensity of the DN relative to the pons were considered positive. Full details of the visible T1-hyperintensity, including the examination in which visible T1-hyperintensity first appeared and the total GBCA dose and time interval between examinations, was recorded for each positive patient.

Thereafter, SI values were determined in operator-defined oval regions-of-interest (ROI) positioned within the DN and pons of all positive patients, as described by Kanda et al. [1]. ROIs were made as large as possible (mean size, 10 mm^2^; range, 6–18 mm^2^) on both the left and right DN with care taken to avoid pulsating vessels (if present) and rim aspects. ROIs in the central pons were adjusted as appropriate to ensure homogeneity. The left and right DN visible on T2-FLAIR images (and, if required, the SWI images) were used to guide accurate ROI positioning on the corresponding unenhanced T1-weighted images.

### Statistical analysis

Unpaired *t* tests were performed to compare the mean DN/Pons (DN/P) SI ratio on unenhanced T1-weighted images at the last MRI examination with the DN/P SI ratio prior to the first contrast-enhanced examination. Separate analyses were performed for patients considered positive for visible T1-hyperintensity and for patients considered negative for visible T1-hyperintensity. The DN/P SI ratio for each patient was calculated after averaging the SI values in the left and right DN. Within each group, separate analyses were performed for all patients combined, for patients that received only gadoterate, for patients that received only gadobutrol, and for patients that received both gadoterate and gadobutrol during imaging follow-up. A significant change in DN/P SI ratio was considered for *p* < 0.05.

## Results

A summary of results is presented in Table 1. A total of 149 patients with confirmed RR-MS underwent two or more MRI exams exclusively at our center with gadoterate meglumine (*n* = 57), gadobutrol (*n* = 63), or both (*n* = 29). These 149 patients included 107 (71.8%) for which visible T1-hyperintensity was not evident on any follow-up MRI exam and 42 (28.2%) for which visible T1-hyperintensity was noted. Highly significant (*p* ≤ 0.0004) quantitative differences in mean DN/P SI ratio were noted for all groups in which visible T1-hyperintensity was noted. Conversely, no significant differences in mean DN/P SI ratio were apparent in any group in which visible T1-hyperintensity was not observed.

**Table 1:** Summary of patients with and without visible T1-hyperintensity of the DN and changes in DN/P SI ratio

Patients	Patient sub-group	Mean age ± SD	M/F	No. of exams	Exam no. at first appearance	Mean interval between exams (months)	Time to first appearance (months)	Mean DN/P before first exam	Mean DN/P before last exam	Unpaired *t* test
Negative	All	51 ± 10.5	26/81	6.75 ± 3.43	n/a	10.6 ± 6.2	n/a	1.025 ± 0.006	1.032 ± 0.005	*p* = 0.323
	Gadobutrol	54 ± 8.5	12/32	7.34 ± 3.82	n/a	10.43 ± 8.3	n/a	1.045 ± 0.008	1.046 ± 0.008	*p* = 0.970
	Gadoterate	47 ± 11.5	8/34	7.21 ± 2.86	n/a	11.3 ± 4.5	n/a	1.001 ± 0.004	1.005 ± 0.003	*p* = 0.4239
	Both	49 ± 6.3	6/15	4.62 ± 4.6	n/a	9.8 ± 6.7	n/a	1.027 ± 0.025	1.06 ± 0.014	*p* = 0.543
Positive	All	50 ± 11.5	8/42	10.21 ± 4.6	4.36 ± 2.5	10.79 ± 6.92	43.48 ± 27.34	1.003 ± 0.004	1.137 ± 0.012	*p* < 0.0001
	Gadobutrol	51 ± 11.2	1/19	11.16 ± 5.4	4.79 ± 2.5	10.9 ± 6.4	48.32 ± 28.7	0.998 ± 0.002	1.153 ± 0.016	*p* < 0.0001
	Gadoterate	42 ± 8.4	6/15	9.07 ± 3.9	3.93 ± 2.4	11.0 ± 8.0	40.0 ± 27.32	1.003 ± 0.004	1.110 ± 0.014	*p* < 0.0001
	Both	60 ± 6.4	1/8	10.13 ± 4.7	4.13 ± 2.8	10.2 ± 6.7	38.5 ± 25.5	1.004 ± 0.011	1.163 ± 0.032	*p* = 0.0004

*n*/*a* not available

The number of administrations per patient in each sub-group is presented in Table 2. A tendency towards a lower number of GBCA administrations per patient was noted for patients with no evidence of visible T1-hyperintensity: 43/107 (40.2%) patients without evidence of visible T1-hyperintensity received five or fewer GBCA administrations compared with only 7/42 (16.7%) patients with evidence of visible T1-hyperintensity. Typically, the first appearance of visible T1-hyperintensity in positive patients occurred at the fourth or fifth follow-up exam, between approximately 3 and 4 years after the initial MRI examination. Few differences were apparent between groups in terms of the time to appearance of the first visible hyperintensity.

**Table 2:** Number of GBCA administrations given to patients with and without visible T1-hyperintensity of the DN

Group	Sub-group	Number of administrations																					No. of patients
		2	3	4	5	6	7	8	9	10	11	12	13	14	15	16	17	18	19	20	21	22	
Negative	All	10	7	14	12	11	13	13	8	7	3	3	–	1	4	–	–	–	–	1	–	–	107
	Gadobutrol	3	2	6	4	5	6	5	3	4	–	2	–	1	2	–	–	–	–	1	–	–	44
	Gadoterate	1	1	5	6	4	7	8	3	2	2	1	–	–	2	–	–	–	–	–	–	–	42
	Both	6	4	3	2	2	–	–	2	1	1	–	–	–	–	–	–	–	–	–	–	–	21
Positive	All	–	2	2	3	3	1	4	4	4	6	3	2	3	1	–	–	1	–	–	2	1	42
	Gadobutrol	–	1	–	1	2	–	2	2	2	3	–	1	2	–	–	–	–	–	–	2	1	19
	Gadoterate	–	1	1	1	1	1	1	1	1	3	3	–	–	1	–	–	–	–	–	–	–	15
	Both	–	–	1	1	–	–	1	1	1	–	–	1	1	–	–	–	1	–	–	–	–	8

**Fig. 1:**
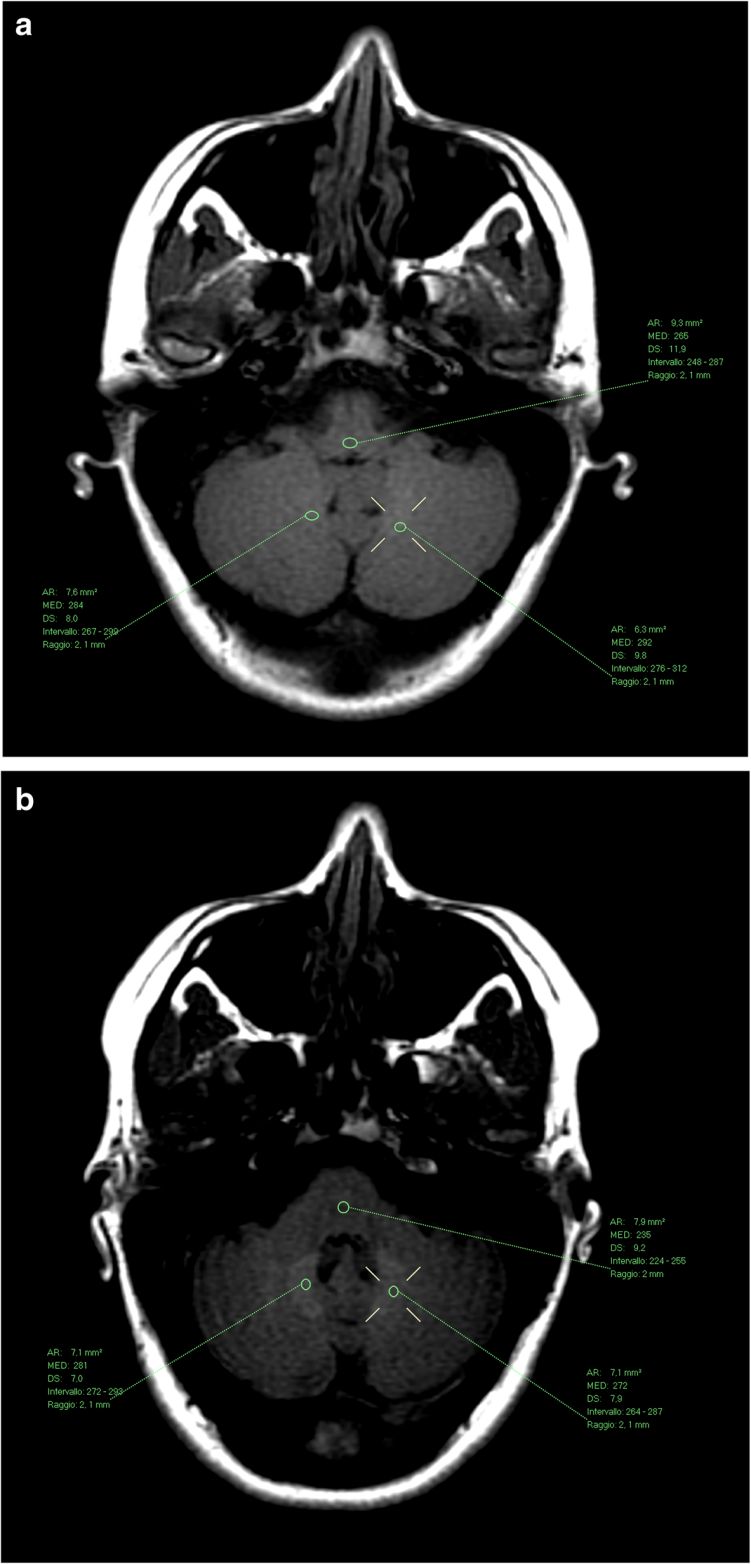
Female patient, 36 years old, before (**a**) and after (**b**) seven administrations of gadoterate meglumine. The mean DN/P SI ratio on T1wSE images increased from 1.09 at the first exam in July 2006 to 1.18 at the last exam in February 2010

**Fig. 2:**
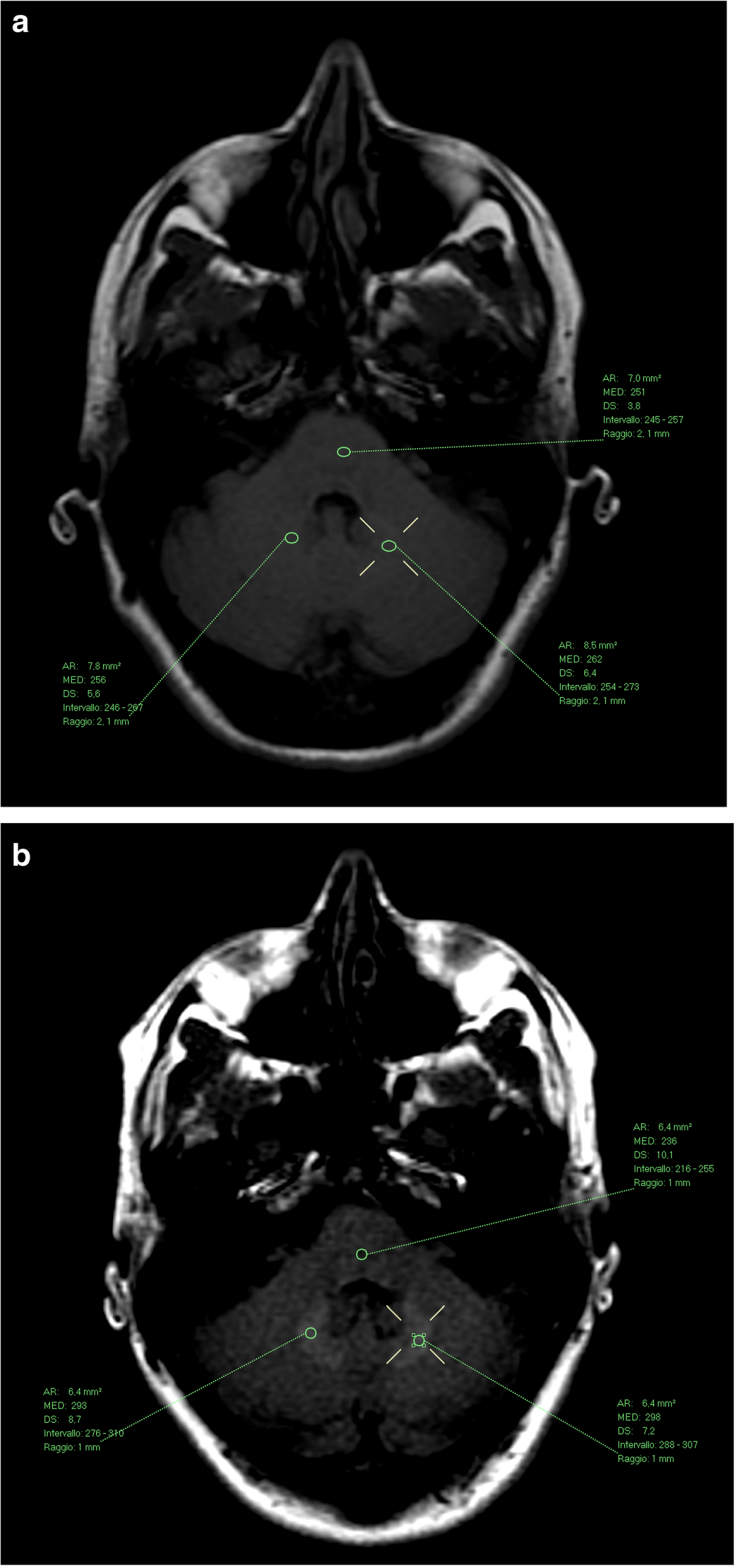
Female patient, 49 years old, before (**a**) and after (**b**) 11 administrations of gadobutrol. The mean DN/P SI ratio increased from 1.03 at the first exam in March 2006 to 1.25 at the last exam in July 2014

**Fig. 3:**
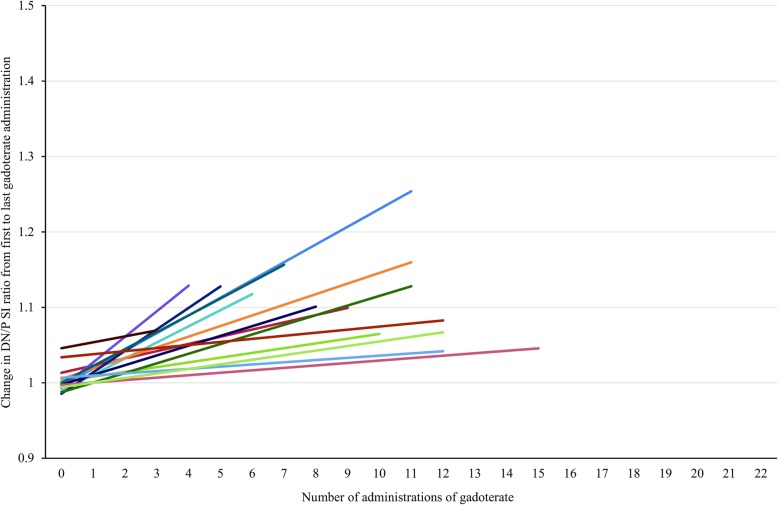
First and last DN/P SI ratios for patients with visible T1-signal changes after two or more administrations of gadoterate meglumine

**Fig. 4:**
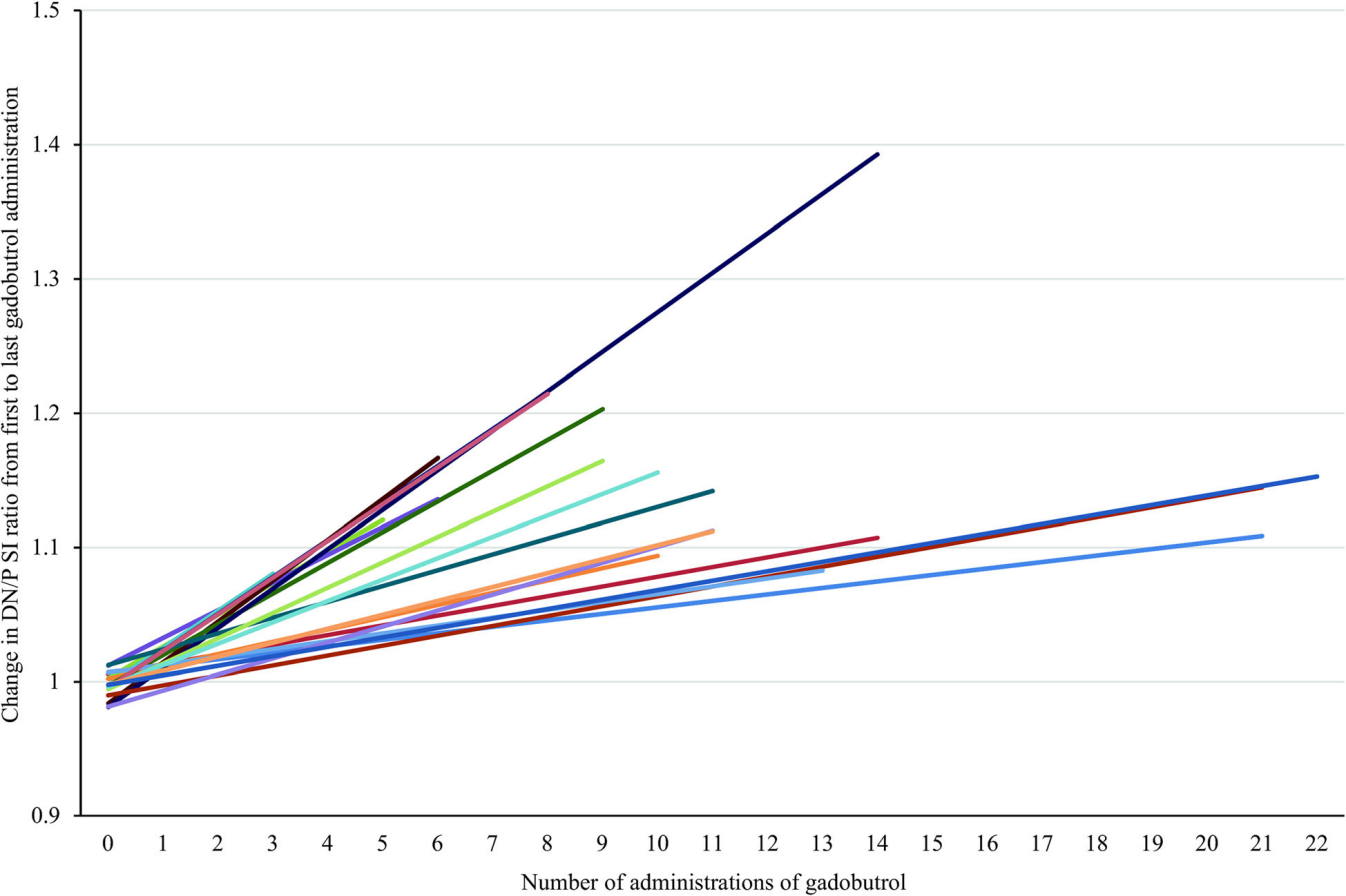
First and last DN/P SI ratios for patients with visible T1-signal changes after two or more administrations of gadobutrol

Examples of the T1-hyperintensity seen after the exclusive administration of gadoterate meglumine and gadobutrol are shown in Figs. 1 and 2, respectively. The increase in DN/P SI ratio from first to last MRI exam in patients with visible T1-hyperintensity is shown in Figs. 3 and 4, respectively.

## Discussion

Although numerous studies have reported quantitative changes in T1-signal in the DN and/or GP following cumulative administration of macrocyclic GBCAs [13–19, 41, 42], comparatively few [15, 16, 18, 42] have reported visible T1-hyperintensity. Those studies that have reported visible T1-signal changes following the administration of macrocyclic GBCAs have largely been overlooked or disregarded, even in official guidelines and recommendations [12]. The results of our study provide clear evidence that T1-signal changes can and do occur in the DN after multiple administrations of the macrocyclic GBCAs, gadobutrol and gadoterate meglumine, and that the appearance of visible T1-hyperintensity is associated with significant increases in DN/P SI ratio.

To note is that not all patients that received multiple administrations of macrocyclic GBCAs demonstrated visible T1-hyperintensity. In common with the findings of many previous studies [3–6], 107/149 (71.8%) patients showed no evidence of visible hyperintensity, even after as many as 20 GBCA administrations. However, it should be noted that patients that demonstrated no signs of visible T1-hyperintensity tended to have received fewer GBCA administrations overall. The appearance of visible T1-hyperintensity in our cohort tended to occur at the 4th or 5th MRI examination whereas approximately 40% of patients negative for visible T1-hyperintensity received five or fewer MRI examinations. It is unclear why some patients demonstrated clear visible T1-hyperintensity of the DN while others did not. Likewise, it is unclear why the relative change in DN/P SI ratio from first to last GBCA administration differed across patients in terms of both magnitude and time course or why the appearance of visible T1-hyperintensity in the DN showed no obvious correlation with quantitative DN/P SI ratio. A recent study by Moreno et al. [18] in melanoma patients referred for serial follow-up gadobutrol-enhanced MRI similarly revealed visible T1-hyperintensity in some, but not all, patients without any association between the DN/P SI ratio and the dose administered or the number of MR examinations. In common with our study, Moreno et al. [18] found a significantly (*p* = 0.024) higher DN/P SI ratio in patients with visible DN T1-hyperintensity than in patients without visible T1-hyperintensity. Other authors have similarly revealed differences over time in terms of DN/P SI ratio with marked differences across patients in terms of the magnitude of changes [43].

Importantly, none of the patients in our cohort had hepatic deficiency or other clinical issues which might explain a differential appearance of DN T1-hyperintensity and none had had previous cerebral neurosurgical treatments or radiation therapy which might explain the appearance of T1-hyperintensity [39, 40, 44–46]. Furthermore, there were no relevant differences between SI measurements in the left and right DN of each patient that might suggest a direct association with MS disease progression. On the other hand, MS is a progressive neurodegenerative disease for which patients undergo frequent follow-up MRI examinations and for which disease progression is a potential confounding variable [8]. An early study reported more frequent T1-hyperintensity of the DN in patients with secondary-progressive MS than in patients with primary progressive disease (46% versus 8%, respectively) and found that this correlated with a higher score on the Expanded Disability Status Scale (EDSS), a higher brain lesion load, and greater tissue loss [47]. However, whereas contrast-enhanced T1-weighted images were acquired with either gadoterate or gadodiamide (Omniscan, GE Healthcare) no information was provided on prior GBCA administrations, so it is difficult to determine whether the observed T1-hyperintensity reflected differences in the stage/extent of disease or differences in the amount/type of GBCA administered. Although some studies [48, 49] have looked to correlate T1-hyperintensity of the DN in patients with MS with loss of verbal fluency and cognition, it is difficult to separate a hypothetic effect of Gd retention from the normal effects of MS progression, especially given the difficulties in finding a matched MS group not exposed to any GBCA. Recent studies by Cocozza et al. [9] and Ackermans et al. [10] suggest that MRI features suggestive of Gd retention do not correlate with EDSS worsening in patients with MS. These findings are supported by studies that have shown no effects of DN T1-hyperintensity on DN tissue integrity [11, 50, 51].

Our findings support those of Stojanov et al. [13] and Splendiani et al. [15] in showing significant increases in DN/P SI ratio following the cumulative administration of macrocyclic GBCAs in patients with RR-MS. Conversely, they are at variance with the findings of others [33–38]. The clear disparity in study findings between research groups highlights the complexity of the issues at hand and the need for carefully controlled large-scale studies to investigate the processes underlying T1-signal changes in these patients. Studies are needed to ascertain whether T1-hyperintensity in the DN is in fact indicative of Gd retention or whether it represents some other process specific to MS and/or other diseases. In the absence of histologic verification of Gd retention, no conclusions can be made as to the processes underlying the T1-signal changes observed in our patient cohort. However, this is the same for all studies that focus solely on observations from imaging studies.

The strength of our study lies in the fact that all patients received only macrocyclic GBCAs and that imaging was performed in a carefully monitored manner exclusively at our center. Therefore, the possibility that patients with visible T1-hyperintensity could have received prior administrations of certain linear GBCAs that are more widely associated with T1-signal changes in the DN can be excluded. Secondly, images from all patients that received at least two macrocyclic GBCA administrations were carefully assessed for visible T1-hyperintensity by two neuroradiologists in consensus. Prior studies to evaluate T1-signal changes have frequently limited the number of patients to a convenient round number (e.g., *n* = 50 [6]), restricted patient inclusion to only those that received a minimum number of GBCA administrations (e.g., *n* = 6), and cited SI values determined by just a solitary reader. Our approach avoided any form of selection bias and was based solely on the visual appearance of the DN relative to the pons. Our approach therefore more typically reflects the assessment process routinely undertaken in clinical practice. Finally, our study included patients with RR-MS evaluated exclusively at our center over an extended period (approximately 13 years). Other studies have drawn conclusions based on very few evaluated patients imaged over a relatively short time frame [37].

A limitation of our study is the absence of an age-matched control group of MS patients that had never been exposed to any GBCA. However, such patients would be extremely difficult to find given that contrast-enhanced MRI is one of the principal imaging techniques for MS patients, both for initial diagnosis and follow-up. A second limitation is that we did not quantify the DN/P SI ratio at every follow-up exam and thus were unable to detail a time course for T1-signal changes in individual patients. However, in quantifying changes in DN/P SI ratio from SI measurements determined prior to the first and last contrast-enhanced exam in each patient, we showed that increases in DN/P SI ratio can and do occur after both gadoterate and gadobutrol and that the changes in T1-signal are variable across patients both in terms of magnitude and time course. Unfortunately, a statistical comparison of these two GBCAs in terms of the changes observed was beyond the scope of this initial study given the relatively small number of positive patients in each group. Finally, we did not attempt to correlate the appearance of visible T1-hyperintensity with cognitive or physical disability as performed elsewhere [9, 10, 49]. Moreover, we did not look at a possible correlation with brain lesion load and tissue loss. This was considered beyond the scope of this initial investigation but should be considered for further large-scale studies in MS patients. Further research should additionally look at changes in R1 relaxation rates which are less prone to potential sources of inaccuracy.

In conclusion, we observed visible T1-hyperintensity of the DN in 42/149 (28.2%) patients with RR-MS who had received at least two administrations of gadobutrol, gadoterate, or both. The appearance of visible T1-hyperintensity correlated with significant increases in DN/P SI ratio from first to last examination.

## Data Availability

All data on patients with confirmed RR-MS are taken from our PACS database.

## Abbreviations

DN: Dentate nucleus
eGFR: Estimated glomerular filtration rate
FLAIR: Fluid-attenuated inversion recovery
FOV: Field of view
GBCA: Gadolinium-based contrast agent
GP: Globus pallidus
MS: Multiple sclerosis
NEX: Number of excitations
P: Pons
ROI: Region of interest
SI: Signal intensity
SWI: Susceptibility weighted images
TE: Echo time
TR: Repetition time
